# Are residential and nursing homes adequately screening overseas healthcare workers?

**DOI:** 10.1186/1756-0500-1-62

**Published:** 2008-08-05

**Authors:** Joanne M Alner, David Hagen, Sue Andrews, Rachel Loveday, Hugo C van Woerden

**Affiliations:** 1 Public Health Department, Wandsworth Teaching PCT, Lupin Ward, Jasmine Tower, Springfield University Hospital, 61 Glenburnie Rd, London SW17 7DJ, UK; 2 Surrey and Sussex Health Protection Unit, College Lane, Chichester, West Sussex, P019 5LX, UK; 3 West Sussex PCT, The Causeway, Goring by Sea, West Sussex, BN16 6BT, UK; 4 Division of Community Health Sciences, St George's, University of London, 6/F, Hunter Wing, Cranmer Terrace, London, SW17 0RE, UK

## Abstract

**Background:**

There has been significant growth in the number of healthcare workers born outside the UK or recruited to the UK from countries with a high prevalence of TB, Hepatitis and other blood borne infections. Government policy recognises the need for occupational health procedures to facilitate treatment for these individuals and to reduce the risk of transmission of disease to patients.

The aim of this study was to undertake a survey of nursing and residential homes in South East England, to assess whether homes had occupational health screening policies for healthcare workers who have originated from overseas, and what level of occupational health screening had been undertaken on these employees.

**Methods:**

An anonymous survey was sent to all 500 homes in West Sussex assessing occupational health practices for "overseas health care workers", defined as health care workers who had been born outside the UK.

**Results:**

Only one employer (0.8%) reported they had an occupational health screening policy specific for healthcare workers who originate from overseas. Over 80% of homes who had recruited directly had no evidence of screening results for HIV, TB, Hepatitis B and C. The commonest countries of origin for staff were the UK, Philippines, Poland, South Africa, Zimbabwe, and India.

**Conclusion:**

This study suggests that screening of overseas healthcare workers is not routine practice for residential or nursing care homes and requires further input from Primary Care Trust's, Health Care Commission, Commission for Social Care Inspection, and Professional bodies.

## Background

There has been significant growth in the number of healthcare workers from other countries to the UK[1]. The proportion from ethnic minority groups has increased from 4% in 2001 to 15% in 2005 and those recruited from overseas had increased from 3% in 2001 to 19% in 2005[2].

The Joint Tuberculosis Committee of the British Thoracic Society code of practice[3] states that new employees from a country where the annual incidence rate is greater than 40 per 100,000 population should be screened either on entry to the country or by their employer.

The BTS code of practice also promotes pre-employment screening of health care workers. This follows changes in the evidence base which suggests that, although in the 1980's the incidence of TB was no higher in healthcare workers than in the general population[4], a more recent study had found a two fold increase in the risk of TB among healthcare workers[5].

A Norwegian study showed that the risk of TB was high in all immigrant groups and higher than those reported in their country of origin. The study acknowledged that this might be due to better detection, or that it could equally be due to the psychological and physiological changes that result from immigration, thus potentially increasing a person's susceptibility to reactivate latent infection[6].

Health care workers in care homes are increasingly being recruited from countries with high prevalence of TB and this raises the possibility of healthcare workers being a significant source of disease for those in their care[7,8].

Department of Health (DH) guidance on control of infection[9] has responded to this issue by indicating that employers are responsible for assessing and responding to this risk.

The DH guidance states that, "Each home should have appropriate policies for the protection of staff through immunisation, training and compliance with health and safety legislation. Such policies should apply to all agency and locum staff and to those on short-term contracts." The guidance also states that, "Appropriate policies should be available to ensure that residents are protected from staff with communicable disease"[9]. Healthcare workers also have a responsibility to provide their employer with documented evidence of their Hepatitis B and TB status[10].

Anecdotal evidence indicated that many residential and nursing homes were not yet compliant with this guidance. Consequently, the aim of this study was to undertake a survey of nursing and residential homes in one county in South East England to assess whether homes had occupational health screening policies for healthcare workers who had originated from overseas and what level of occupational health screening had been undertaken on these employees. A cross sectional survey was considered an appropriate methodology to answer this question.

## Methods

A survey was posted to all 500 residential and nursing homes, referred to collectively as care homes, within one county in the South East of the UK during the winter period of 2005/2006.

The survey was addressed to the manager of the home and included an explanation of the purpose of the survey. A self-addressed enveloped was provided. To encourage a higher response rate the questionnaires could be returned anonymously.

Members of the local Health Protection Team piloted the survey. These individuals had prior nursing experience and routinely communicated with managers of care homes in the county.

The survey of the home requested information on staff numbers, gender, overseas health care workers' country of origin, recruitment methods, occupational health policy, and availability of health screening blood tests. An overseas healthcare worker was defined as, "an individual who was not born in the UK".

Due to time and cost restraints, a reminder letter and questionnaire were not sent out.

## Results

One hundred and two questionnaires were returned; a 20% response rate, providing data on 475 overseas healthcare workers (HCW). A range of care home providers responded, including both public and private sector care homes, and small and large employers. A small number of questionnaires were returned as "not applicable", this was in part due to the use of the term "nurse" in place of health care worker in parts of the questionnaire. Where possible contact was made with these care homes and when appropriate the questionnaire was completed over the phone.

Only one employer (0.8%) reported they had an occupational health screening policy specific for healthcare workers who originate from overseas currently in place. The survey revealed that 21% (475/2299) of healthcare workers were considered to have originated from overseas; a third of the male workforce originated from overseas (131/407). On average, each home employed five 'overseas healthcare workers'. The most common country of origin recorded was the Philippines (Table 1).

**Table 1:** Country of origin of overseas Health Care Worker's employed in Care Homes in a county in South East England during 2006

**Country of origin**	**Proportion of employed staff**
Philippines	24/186 (13%)
Poland	20/186 (11%)
South Africa	19/186 (10%)
Zimbabwe	19/186 (10%)
India	15/186 (8%)

61% (60/101) of homes reported that they currently employed 'overseas healthcare workers' and 35% (35/101) reported that they had never recruited such staff. Of those who had recruited overseas staff, 27% (28/101) had recruited via a nursing agency, 2% (2/101) used agencies based outside the UK and 48% (48/101) recruited these staff directly. 'Overseas healthcare workers' recruited locally were presumably residing within the UK before they were employed. 47% (14/30) of homes who used the services of a nursing agency believed the agency conducted some type of health screening but the majority did not have evidence of the results of this process.

Over 80% of homes who had recruited directly had no evidence of screening results for HIV, TB, Hepatitis B and C (Table 2). Nursing agencies were slightly more likely to gain this evidence than those who directly recruited. Apart from for HIV testing, the difference was not statistically significant.

**Table 2:** Proportion of overseas Health Care Worker's employed in Care Homes in a county in South East England who had evidence of Health Screening for a range of relevant conditions during 2006

**Test**	**Directly recruited by the home**	**Recruited via a nursing agency**
HIV	6%	27%
Hepatitis B	10%	27%
Hepatitis C	4%	13%
Chest x-ray to exclude TB	8%	10%
Mantoux or Heaf test to exclude TB	6%	17%

Only 9% (09/101) of homes reported their organisation provided occupational health services. An additional 10% (10/101) appeared to rely on the GP of the employee to provide occupational health advice. The majority of the respondents (79%) did not reply to this question.

## Discussion

The most concerning findings of this study was the lack of relevant occupational health pre-employment screening policies. The majority of those who responded did not obtain evidence of relevant vaccination or ensure the absence of infectious disease prior to employment of individuals in their organisation. Managers in the homes frequently stated that they were not aware of any increased risk from 'overseas health care workers' and that they did not want to appear to be discriminating against individuals from overseas by making their occupational health screening more stringent.

Obtaining a vaccination and immunisation history should be the minimum requirement of any occupational health policy and should be applied to all healthcare workers. This, if applied universally, would prevent employers from fearing they are discriminating against overseas healthcare workers. Coker[11] suggests these procedures should carry the ethos of a "welcome health check", and should be provided alongside clear access to primary care services and occupational health support.

Ten percent of the responses considered the GP to be the occupational health provider, relying on the health screen offered at registration with a GP to identify any health concerns. However, this is not the purpose of a GP health screen and this relies on all new entrants to the UK registering with a GP. The Joseph Rowntree Foundation[12] reported that there is lack of understanding among new immigrants about how to access healthcare. This may be due to healthcare systems being different between the UK and an individual's home country and/or employees' fears of being returned home if found to be unwell.

Immigrants from countries with a TB Incidence rate of over 40/100,000 are considered to be at sufficient risk of TB to merit additional health screening (see Table 3). As shown in Table 1, most of the health care workers identified in this study were from such countries.

**Table 3:** Published incidence rates for TB in countries relevant to this study [13]

**Country**	**Incidence Rate**
Philippines	293/100,000
Poland	29/100,000
South Africa	718/100,000
Zimbabwe	674/100,00
India	168/100,000
UK	12/100,000

The countries of origin of the individuals reported in this study are similar to those suggested by Buchan[1] and the analysis of the overseas admissions to the UKCC during 2000–2001. The UKKCC study showed a higher proportions of Polish health care workers, which may have been related to immigration from Poland in the immediate aftermath of Poland's entry to the EU in 2002.

### Limitations of the study

The 20% response rate was disappointing and we would have wished for this to be higher to increase the generalisability of the findings.

The use of the term "nurse" in the survey did cause misunderstanding with some responders. This was addressed wherever possible, and where a telephone call indicated that the home was appropriate for inclusion in the study. Another possible barrier to achieving a higher response rate could have been that a high level of goodwill was needed to complete the survey. Managers needed to be willing and able to share sensitive organisational information with an outside agency about recruitment practices. Although there was the option to be anonymous, this may have deterred managers from completing the survey and the authors are grateful to those who were willing to respond.

Another limitation of this study is that the focus on infectious diseases, from a public health protection perspective, may have distracted from the importance of other pre-employment health screening criteria. It should be acknowledged that there are additional implications of inadequately health screening of new employees, in areas such as psychological profiles, or history of substance misuse, which this study did not aim to assess.

## Conclusion

This study indicates that screening of healthcare workers is not routine practice for most nursing and residential care homes in the UK and needs to be improved. The vulnerable client group, which nursing and residential homes serve, can only be fully protected if both the employee and employer are more open, vigilant, and act upon any issues that arise.

Employers should be screening and asking clear questions of all health care workers, regardless of nationality. Equally, health care workers should ensure that they act responsibly, provide relevant medical history, and act upon any findings from occupational pre-employment screening.

Professional bodies which regulate the registration of staff, Primary Care Trusts, the Care Standards Agency, and Commission for Social Care Inspection also have a role in ensuring that monitoring processes identify whether effective policies and processes are in place and being effectively implemented.

## Authors' contributions

JA: led on the design and co-ordination of the study, analysis and interpretation of the data and participated in the drafting of the manuscript

DH: conceived of the study, and participated in its design and coordination and revising of the manuscript critically for important intellectual content

SA: conceived of the study, participated in its design and coordination and revising of the manuscript critically for important intellectual content

RL: conceived of the study, and participated in its design and coordination

HvW: led on drafting the manuscript

All authors read and approved the final manuscript.
